# Multiple Imputation for Partial Recording Periodontal Examination Protocols

**DOI:** 10.1177/23800844221143683

**Published:** 2023-01-16

**Authors:** J.S. Preisser, T. Shing, B.F. Qaqish, K. Divaris, J. Beck

**Affiliations:** 1 Department of Biostatistics, Gillings School of Global Public Health, University of North Carolina at Chapel Hill, Chapel Hill, NC, USA; 2 Division of Pediatric and Public Health, Adams School of Dentistry, University of North Carolina at Chapel Hill, NC, USA; 3 Department of Epidemiology, Gillings School of Global Public Health, University of North Carolina at Chapel Hill, Chapel Hill, NC, USA; 4 Division of Comprehensive Oral Health/Periodontology, Adams School of Dentistry, University of North Carolina at Chapel Hill, Chapel Hill, NC, USA

**Keywords:** biostatistics, chronic disease surveillance, computer simulation, dental public health, epidemiology, periodontal disease(s)/periodontitis

## Abstract

**Aim:**

Partial-mouth recording protocols often result in underestimation of population prevalence and extent of periodontitis. We posit that multiple imputation of measures such as clinical attachment loss for nonselected tooth sites in partial-mouth samples can reduce bias in periodontitis estimates.

**Methods:**

Multiple imputation for correlated site-level dichotomous outcomes in a generalized estimating equations framework is used to impute site-level binary indicators for clinical attachment loss exceeding a fixed threshold in partial-mouth samples. Periodontitis case definitions are applied to the imputed “complete” dentitions, enabling estimation of prevalence and other summaries of periodontitis for partial-mouth samples as if for full-mouth examinations. A multiple imputation-bootstrap procedure is described and applied for point and variance estimation of these periodontitis measures. The procedure is evaluated with pseudo-partial-mouth samples based on random site selection protocols of 28 to 84 periodontal sites repeatedly generated from full-mouth periodontal examinations of 3,621 participants in the 2013 to 2014 National Health and Nutrition Examination Survey (NHANES) survey.

**Results:**

Multiple imputation applied to partial-mouth samples overestimated periodontitis mean extent, defined as the number of sites with clinical attachment loss 3 mm or greater, by 9.5% in random site selection protocols with 84 sites and overestimated prevalence by 5% to 10% in all the evaluated protocols.

**Conclusions:**

In the 2013 to 2014 NHANES data, multiple imputation of site-level periodontal indicators provides less biased estimates of periodontitis prevalence and extent than has been reported from estimates based on the direct application of full-mouth case definitions to partial-mouth samples. Multiple imputation provides a promising solution to the longstanding, vexing problem of estimation bias in partial-mouth recording, with potential application to a wide array of case definitions, periodontitis measures, and partial recording protocols.

**Knowledge Transfer Statement:**

Partial-mouth sampling, while a resource-efficient strategy for obtaining oral disease estimates, often results in underestimation of periodontitis metrics. Multiple imputation for nonselected periodontal sites produces pseudo-full-mouth data sets that may be analyzed and combined to produce estimates with small bias.

## Introduction

Periodontal disease surveillance is impeded by changing case definitions and the use of a full-mouth periodontal examination (FMPE), which may be burdensome, lengthy, and costly. In studies of periodontitis in adults, the gold standard FMPE records clinical variables at 6 sites per tooth, for up to 28 teeth (excluding third molars), which can take 40 min to complete. Partial-mouth recording protocols (PRPs) provide substantial time- and cost-savings. In a partial-mouth exam, a subset of tooth sites is examined using either random site selection methods (RSSMs; Beck et al. 2006; Preisser et al. 2017) or fixed site selection methods (FSSMs; Alexander 1970; Mills et al. 1975; Fleiss et al. 1987). A major obstacle to the use of PRPs is that the standard method that applies a full-mouth periodontitis case definition directly to partial-mouth samples systematically underestimates periodontitis prevalence (Kingman and Albandar 2002; Susin et al. 2005; Tran et al. 2014).

Under the standard method, PRPs that select teeth (or sites) with the most disease tend to have higher sensitivity and less underestimation bias than PRPs that identify a “representative” selection of sites such as RSSMs (Beck et al. 2006) or Ramfjord teeth (Ramfjord 1959). The choice of PRPs that select teeth most susceptible to periodontal disease is routinized in a population ranking method (Alshihayb et al. 2022) based on a single cardinal measure: clinical attachment loss (CAL) or probing depth (PD). Similarly, case definitions proposed by the Group C Consensus Report of the Fifth European Workshop on periodontology (Tonetti et al. 2018) and the Centers for Disease Control and Prevention in conjunction with the American Academy for Periodontology (CDC/AAP) restrict consideration to interproximal (IP) sites (Eke et al. 2012), known to have relatively high levels of disease. While there has been progress in the clinical classification of periodontitis using PRPs (Alshihayb et al. 2022; Botelho et al. 2020), less progress has been made on the reliable use of PRPs in estimating periodontitis prevalence and extent.

Problematically, the underestimation of mean extent and prevalence of periodontitis by the standard method is frequently severe. Extent is the number of sites in an individual mouth exceeding an established threshold, that is, ≥ CAL 3 mm (i.e., CAL3+) or ≥ PD 4 mm (i.e., PD4+) (Heaton, Sharma, et al. 2018), whereas prevalence is the proportion of individuals in a population who fulfill a certain criterion (i.e., case definition) defined a priori. In a study of 10,680 participants from National Health and Nutrition Examination Survey (NHANES) cycles 2009 to 2014 (Alshihayb et al. 2022), PRPs examining all 6 sites from half-mouth protocols resulted in mean extent estimates with 45% to 55% underestimation relative to full-mouth extent, and the population ranking method that selects the 14 most diseased teeth had bias ranging from 25% to 38%. Eke et al. (2010) found that the NHANES III and NHANES 2001 to 2004 protocols underestimated the prevalences of moderate or severe periodontitis by the CDC/AAP case definitions by more than 50%; case definitions based on 1 or more sites with CAL meeting 3-mm or 6-mm thresholds resulted in underestimation of prevalence between 30% and 40%. This substantial bias has limited the use of PRPs, which ceased to be used by NHANES in 2009.

The excessive bias in these studies results from the direct application of full-mouth case definitions for periodontitis to partial-mouth samples. Assuming no measurement error at the site level, this approach systematically underestimates prevalence and extent because subjects with no diseased sites in the full mouth are always classified correctly as having no disease (i.e., specificity is 100%), whereas subjects with diseased sites could have zero such sites selected and thus be classified as without periodontitis (i.e., sensitivity is less than 100%). A new approach is needed to reduce estimation bias when using PRPs for periodontitis surveillance.

Within a general framework for well-defined estimands (i.e., the target parameter in the population of interest based on a FMPE), this article aims to show that multiple imputation (MI) of nonselected tooth sites in PRPs can provide population periodontitis estimates of extent and prevalence with small bias. Imputation fills in missing data for nonselected sites to create pseudo-“complete” clusters (individual dentitions) mimicking FMPEs. Single imputation (i.e., “filling in” missing data with 1 set of plausible values) fails to account for the uncertainty of the imputation model. MI overcomes this limitation by producing multiple “complete” data sets, each consisting of pseudo-full dentitions for all individuals in the sample, that differ with respect to their imputed values. When the model generating the imputations is correct, the distributions across these data sets of the imputed values for each missing datum implicitly reflect appropriate estimates of both the missing values and the underlying random variability. MI has rules for combining the multiple estimates of a quantity, such as prevalence or extent from the “complete” data sets, into a single overall estimate of that quantity and for pooling variability of the individual estimates between and within imputations into an overall variance of the overall estimate (Shafer and Graham 2002). In this article, the proposed MI methods are evaluated using repeated RSSMs generated from the FMPE data in the NHANES 2013 to 2014 study population.

## Methods

### A Multiple Imputation Estimator for the Mean Number of Diseased Sites in PRPs

In a population, the goal of periodontal disease surveillance is to estimate the mean of some individual-level characteristic derived from site-level periodontal information. While the quantification or identification of disease often incorporates multiple types of periodontal measures (e.g., CAL and PD), for clarity, we consider a single cardinal measure of periodontal disease, CAL, that allows direct comparison to published results for extent (Alshihayb et al. 2022). Using CAL, 3 types of disease metrics are common. The first quantifies disease severity as the mean CAL measurement for sites in the mouth. Severity is not considered further since use of PRPs, especially RSSMs, typically estimates it with small bias (Brown and Löe 1993; Kingman et al. 2008). The second and third metrics, extent and prevalence, depend upon site-level threshold indicators (e.g., CAL3+). Note that prevalence, which is the proportion of individuals with disease according to case classification, is a mean of 0s (disease absent) and 1s (disease present). Thus, the presentation of the MI procedure described herein for population means encompasses both prevalence and extent.

### Periodontal Disease Estimands

It is important to define the population quantity that is being measured—the estimand. In a population of *K* individuals, the *i*th individual’s disease is quantified by a summary statistic $S_{i,n_{i}}$ that is an aggregation of site-level variables from the individual’s *n_i_* tooth sites where max{n_i_} = 28 teeth × 6 sites per tooth = 168, excluding third molars. Specifically, let *Y_ij_* be a binary indicator for exceeding the threshold measure (e.g., CAL3+) for the *i*th subject at the *j*th site, where *j* = 1,. . .,*n_i_*. The number of periodontal sites with CAL3+ (extent) is given by $S_{i,n_{i}}=\overset{n_{i}}{\underset{j=1}{\sum }}Y_{ij}$ , whereas, for prevalence, $S_{i,n_{i}}=I(\overset{n_{i}}{\underset{j=1}{\sum }}Y_{ij}\geq h)$ with indicator function I (·) equal to 1 if the condition inside the brackets is true and 0 otherwise. When *h* = 1, the case definition is 1 or more sites with CAL3+, and when *h* = 2, it is 2 or more sites affected. The estimand is the population average (or expectation, *E*) of the summary statistic over all individuals in the population, expressed $E(S_{i,n_{i}}),$ which depends on the empirical population distribution of the number of teeth. More precisely, accounting for heterogeneity due to the number of teeth in the mouth, the estimand is defined by double expectation, $E_{n_{i}}\left\{E\left[\overset{n_{i}}{\underset{j=1}{\sum }}Y_{ij}|n_{i}\right]\right\}$ , where the outer expectation is a weighted average over the patients with respectively 1 to 28 remaining teeth, and *n_i_* are the corresponding multiples of 6. The inner expectation applies to subpopulations of individuals with the same number of teeth and the outer expectation averages over the subpopulations. Thus, $\tau \left(n_{i}\right)=E\left[\overset{n_{i}}{\underset{j=1}{\sum }}Y_{ij}|n_{i}\right]$ is the subpopulation mean number of sites with CAL3+ among individuals with *n_i_* periodontal sites, and the extent estimand averaged over the subpopulations is $\tau_{A}$ = $E_{n_{i}}\left\{\tau (n_{i})\right\}.$ Likewise, $\pi \left(n_{i}\right)=P(S_{in_{i}}=1|n_{i})$ is the proportion of individuals in the subpopulation with *n_i_* tooth sites satisfying the case definition and $\pi_{A}=E(\pi (n_{i}))$ is the average prevalence. The subscript *A* denotes averaging over the subsets of individuals with different numbers of remaining non–third molar teeth. The following presentation focuses on $\tau_{A}$ , with results for *πA* also included.

### Full-Mouth Estimators

As MI creates “complete” full-mouth data, estimators of $\tau_{A}$ and *πA* for FMPEs are introduced first. An FMPE estimator for extent of CAL3+ based on a random sample of *K* individuals, possibly with unequal probabilities of selection, is the weighted average number of sites with CAL3+:

$${\hat{\tau }}_{A,f}=\frac{\sum_{i=1}^{K}w_{i}S_{i,n_{i}}}{\sum_{i=1}^{K}w_{i}},$$

with sampling weights *w_i_* equal to the inverse probability of selection and $S_{i,n_{i}}=\overset{n_{i}}{\underset{j=1}{\sum }}Y_{ij}$ . Variance estimates are computed using Taylor series linearization to account for unequal weighting. In a large simple random sample of individuals from the population, $w_{i}=1$ gives asymptotically unbiased inference, whereas, for complex sample surveys such as NHANES, individual weights reflecting the unequal selection probabilities of each member of the sample (“design weights”) are used. The same general formula above applies for the FMPE prevalence estimator πˆ*A,f*, where $S_{i,n_{i}}=I(\overset{n_{i}}{\underset{j=1}{\sum }}Y_{ij}\geq h)$ .

### Partial-Mouth Estimators

Consider a random sample of *K* individuals who undergo a PRP of *m* < 168 randomly selected tooth sites; when mouths have fewer than *m* sites, $m_{i}=\min(m,n_{i})$ tooth sites are selected. To estimate $\tau_{A}$ from a PRP, we assign a value to $S_{i,n_{i}}$ for each sampled individual by imputing *Y_ij_* for nonselected tooth sites. An estimator of $\tau_{A}$ is the inverse probability of selection weighted sample average number of periodontal sites with CAL3+:

$${\hat{\tau }}_{A}=\frac{\sum_{i=1}^{K}w_{i}(\sum_{j=1}^{m_{i}}Y_{ij}+\sum_{j=m_{i}+1}^{n_{i}}{\hat{Y}}_{ij})}{\sum_{i=1}^{K}w_{i}},$$

where ${\hat{Y}}_{ij}$ is the imputed indicator variable of CAL3+ for the *n_i_ – m_i_* nonselected sites with selected sites first in the ordering, without loss of generality. Similarly, the PRP estimator of *πA* depends on the case classification applied to the imputed “complete data set” of site-level indicators, expressed ${\hat{S}}_{i,n_{i}}=I\left(\left[\overset{m_{i}}{\underset{j=1}{\sum }}Y_{ij}+\overset{n_{i}}{\underset{j=m_{i}+1}{\sum }}{\hat{Y}}_{ij}\right]\geq h\right).$ Tooth-site CAL measurements missing for technical reasons were less than 1% at each tooth site in the NHANES data and were imputed along with nonselected sites.

### Multiple Imputation Method

The MI method consists of an imputation and a calculation stage. First, the imputation model specifying the first and second moments (i.e., site-level probabilities and pairwise correlations) must be identified and estimated using partial-mouth data. During the imputation stage, site-level binary variables are imputed recursively as ${\hat{Y}}_{ij}$ from Bernoulli distributions defined by conditional probabilities that match their relative frequencies and pairwise correlations, thereby generating MI replicates with “complete” full-mouth data. In the calculation stage, established rules (Rubin 1987) are used to obtain overall cluster-level estimates; bootstrapping is applied to each imputation replicate to compute within-imputation variances. This adheres to the MI followed by bootstrapping (MI-boot) approach described by Schomaker and Heumann (2018). Bootstrap estimation has proven to be a useful tool for obtaining standard errors when analytic solutions are not available. Among methods that combine bootstrapping with MI, MI-boot has been shown to be reasonable and computationally feasible (Schomaker and Heumann 2018; Brand et al. 2019; Bartlett and Hughes 2020). The proposed method has 3 steps:

**Step 1.** Identify the imputation model for the site-level multivariate binary data incorporating assumptions about the level of disease across tooth sites and the pattern (correlation) among site pairs. Considering that RSSM data are missing completely at random, we recommend analysis with generalized estimating equations (GEEs; Prentice 1988) that use the partial-mouth data to flexibly and simultaneously fit the 2 regressions of the imputation model: one for the marginal mean (i.e., probability of disease at the tooth site level [with regression coefficients β]) and the second for the within-cluster (mouth) pairwise correlation structure (with regression coefficients α). The log of the number of teeth is included as a mouth cluster-level covariate in both regressions. The GEE analysis of the paired regressions is performed using the SAS macro GEECORR (Shing et al. 2021). The resulting parameter estimates $\hat{\theta }=(\hat{\beta },\hat{\alpha })$ and their estimated variance matrix are used in step 2.

**Step 2.** Impute disease status for “missing” nonselected tooth sites creating *M* “complete” data sets. To create each of the *M* imputed replicate data sets, a random draw is made from $\theta^{*}\sim MVN(\hat{\theta },V(\hat{\theta })),$ the posterior predictive (multivariate normal) distribution of the parameters (i.e., the conditional distribution of the parameter given a relatively vague prior distribution and the observed data). This allows for between-imputation variability as each imputation replicate will have different mean and pairwise correlations. Specifically, the drawn $\theta^{*}=$ ( $\beta^{*},\alpha^{*}$ ) determines the marginal means and correlation matrix (mi*, Ri*) from the conditional linear family (CLF) of joint distributions for multiple correlated binary variables (Qaqish 2003; Preisser and Qaqish 2014) providing a distinct statistical distribution model (SDM) for the clustered site-level disease indicators for each individual based on predictive covariates in the pair of regression equations. The missing site indicators $Y_{ij},j=m_{i}+1,\ldots ,n_{i}$ are randomly imputed as ${\hat{Y}}_{ij}$ by random sampling from the SDM for each individual (see Appendix for details). A SAS macro for performing the imputations is found on GitHub: https://github.com/tshing17/CLF-Imputation-SAS-Macro.

**Step 3.** Calculate estimates based on MI combining rules. During the calculation phase of the method, extent and corresponding variances are estimated using established rules (Rubin 1987). The MI estimate of extent is the average of estimates across *M* imputed samples:

$${\bar{\tau }}_{A}=\frac{1}{M}\overset{M}{\underset{l=1}{\sum }}{\hat{\tau }}_{Al},$$

where ${\hat{\tau }}_{Al}$ is the estimate for the *l*th imputation replicate. The imputation-based variance is

$${\hat{V}}_{A}=V_{AW}+(1+\frac{1}{M})V_{AB},$$

where $V_{AB}$ is the between-imputation variance of the estimates over all *M* imputed data sets:

$$V_{AB}=\frac{\sum_{l=1}^{M}{\left({\bar{\tau }}_{A}-{\hat{\tau }}_{Al}\right)}^{2}}{M-1},$$

and *V*_AW_ is the average within-imputation variance of the estimates

$$V_{AW}=\frac{1}{M}\overset{M}{\underset{l=1}{\sum }}{\hat{V}}_{Al},$$

where ${\hat{V}}_{Al}$ is the bootstrap variance estimate of ${\hat{\tau }}_{Al}$ computed using the MI-boot method described by Schomaker and Heumann (2018) and Bartlett and Hughes (2020). To compute ${\hat{V}}_{Al},$ *B* bootstrap samples are drawn for each of the *M* imputed data sets. Bootstrap estimates are computed for each bootstrap sample of the *l*th imputation replicate, denoted ${\hat{\tau }}_{Al,b}$ , where b=1,…,B;l=1,…,M . Then, for the *l*th replicate, the variance of ${\hat{\tau }}_{Al}$ is computed as

$${\hat{V}}_{Al}=\frac{\sum_{b=1}^{B}{\left({\hat{\tau }}_{Al,b}-{\hat{\tau }}_{Al,boot}\right)}^{2}}{B-1},$$

where ${\hat{\tau }}_{Al,boot}=B^{-1}\overset{B}{\underset{b=1}{\sum }}{\hat{\tau }}_{Al,b}$ is the average estimate of all bootstrap samples for the *l*th replicate.

### Evaluation

NHANES 2013 to 2014 periodontal examination data were used to illustrate the utility of MI for RSSMs in the evaluation of periodontitis estimators relative to gold standard FMPE estimators. We considered the mean number of periodontal sites with CAL3+ (extent) and 2 site-threshold periodontitis case definitions for prevalence: 1 or more sites with CAL3+ and 2 or more sites with CAL3+.

### RSSM Sampling of NHANES 2013–2014 Full-Mouth Periodontal Exam

Partial-mouth extent and prevalence estimators were evaluated by inducing 4 RSSMs from the NHANES 2013 to 2014 FMPE data. For each RSSM, participants (clusters) were resampled with replacement to generate 500 samples of 3,621 clusters. Then, 28, 36, 42, or 84 tooth sites were randomly selected per RSSM protocol (excluding third molars). Sites from missing teeth (i.e., nonexistent tooth sites) were excluded; however, unmeasurable sites were eligible for selection. For comparison, FMPE estimators were also evaluated by generating 500 with-replacement samples of 3,621 clusters (the number of participants in the study), by using data from all tooth sites.

### Imputation Model for NHANES 2013–2014 RSSM Samples

The MI procedure was applied to each RSSM evaluation sample. The SDM used for imputation of missing dichotomous CAL3+ values consisted of a logistic regression for the marginal mean (probability of a tooth site being affected) and a linear model for within-mouth correlations among site pairs estimated jointly by GEE (Prentice 1988). Predictors in the mean model consisted of indicators for each of the 6 tooth sites (distal buccal, buccal, mesiobuccal, mesiolingual, lingual, and distal lingual), sextant tooth location (maxillary right posterior, maxillary anterior, maxillary left posterior, mandibular left posterior, mandibular anterior, and mandibular right posterior), the log number of teeth, and categorical age (30–39, 40–49, 50–59, 60–69, 70+ y). The correlation model consisted of indicators for site pairs located on the same tooth, pairs of sites located on different teeth, pairs of adjacent sites that share the same IP space, pairs of adjacent sites that do not share the same IP space for sites, and pairs of sites on teeth that are directly above and below each other with 1 tooth on the maxillary jaw and 1 tooth on the mandibular jaw.

For each RSSM evaluation sample, the mean and correlation models were fitted to produce $\hat{\theta }=(\hat{\beta },\hat{\alpha })$ and their variance that, as described above, defined the mean and variance of the posterior predictive distribution. At the imputation stage, based on replicate random draws of θ^*^, *M* = 25 “complete” data sets were created using the modified CLF algorithm. The imputation-based estimate ${\bar{\tau }}_{A}$ and its ${\hat{V}}_{A}$ were computed as described above; *B* = 200 bootstrap samples of the “complete” clusters were used to compute the variance of the estimate for the *l*th imputation replicate (Schomaker and Heumann 2018; Bartlett and Hughes 2020).

### Metrics for the Comparison of MI and Full-Mouth Estimates

The MI-boot estimators were compared to their respective FMPE estimators using evaluation metrics similar to bias and efficiency; for convenience, these are referred to as such. Since the true value of $\tau_{A}$ is unknown, the full-mouth estimate ${\hat{\tau }}_{A,f}$ was used as the gold standard. In particular, the percent relative bias of ${\bar{\tau }}_{A}$ was calculated as the scaled difference between the average of the 500 replicate MI-boot estimates and the full-mouth estimate (Table 1). The amount of information lost by using an RSSM relative to an FMPE was estimated with percent relative efficiency calculated as the ratio of the full-mouth variance estimate to the average of the 500 replicate MI-boot estimates. Finally, the percent relative bias of MI-boot variance estimator ${\hat{V}}_{A}$ relative to the Monte Carlo variance of the MI-boot estimates was also computed.

**Table 1.** Evaluation Metrics for the MI-boot Method for the Mean Extent of Periodontitis Using 500 Simulations of Random Partial-Mouth Periodontal Examinations from NHANES 2013–2014.

Metric	Formula	Where
Percent relative bias of the MI-boot extent estimator	$\frac{1}{500}\overset{500}{\underset{r=1}{\sum }}\left[\frac{\left({\bar{\tau }}_{A,r}-{\hat{\tau }}_{A,f}\right)}{{\hat{\tau }}_{A,f}}\right]\times 100$	${\bar{\tau }}_{A,r}$ is the MI-boot estimate of the *r*th replicate and ${\hat{\tau }}_{A,f}$ is the full-mouth estimate.
Percent relative efficiency of the MI-boot estimator	$\frac{{\hat{V}}_{A,f}}{\frac{1}{500}\sum_{r=1}^{500}\left[{\hat{V}}_{A,r}\right]}\times 100$	${\hat{V}}_{A,r}$ is the MI-boot variance estimate of the *r*th replicate and ${\hat{V}}_{A,f}$ is the variance of the full-mouth estimator.
Percent relative bias of the MI-boot variance estimator	$\frac{\sum_{r=1}^{500}\left[{\hat{V}}_{A,r}/500\right]-{\hat{V}}_{A,MC}}{{\hat{V}}_{A,MC}}\times 100$	${\hat{V}}_{A,MC}$ is the Monte Carlo variance estimator below.
Monte Carlo variance estimate of the MI-boot estimator	${\hat{V}}_{A,MC}=\overset{500}{\underset{r=1}{\sum }}{\left[{\bar{\tau }}_{A,r}-\overset{500}{\underset{r=1}{\sum }}{\bar{\tau }}_{A,r}/500\right]}^{2}$ /499	

Extent is defined as the number of tooth sites with CAL3+.

CAL, clinical attachment loss; MI, multiple imputation.

## Results

### Evaluation Results for the Mean Number of Sites with CAL3+

**Figure 1.**
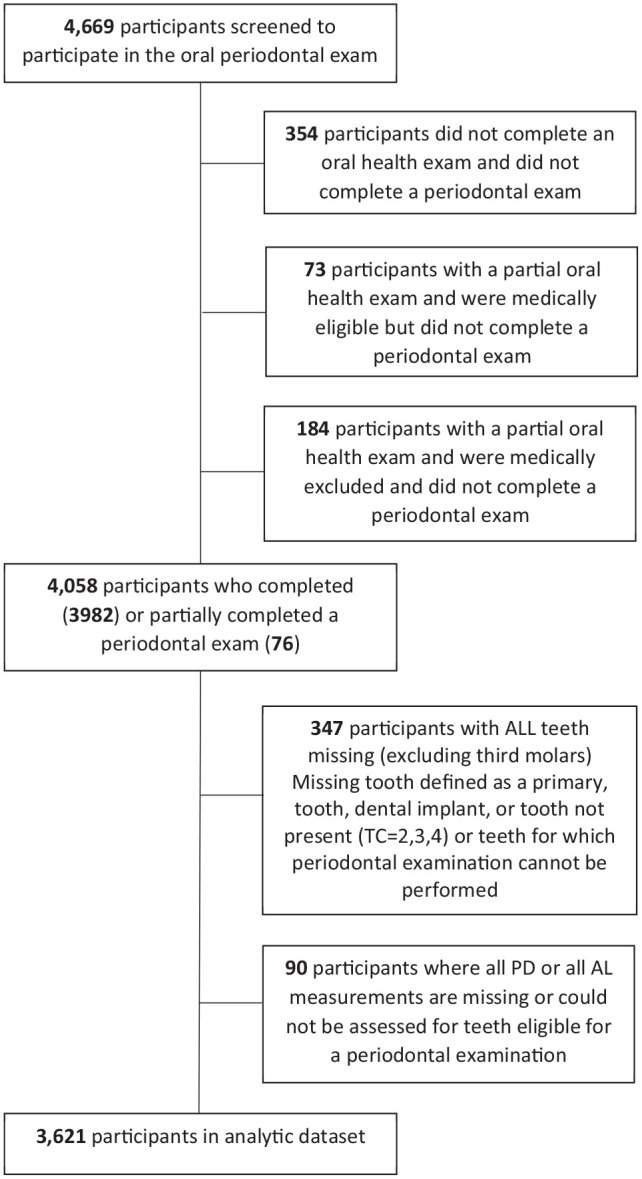
The selection of National Health and Nutrition Examination Survey (NHANES) 2013 to 2014 study participants.

**Figure 2.**
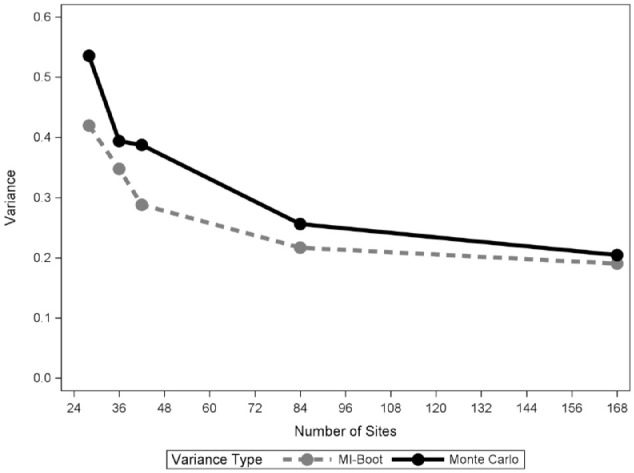
Average MI-boot and gold standard Monte Carlo variance estimates for the mean number of tooth sites with CAL3+ according to number of sites in four random site selection methods. CAL, clinical attachment loss; MI, multiple imputation.

The analytic data consisted of 3,621 participants with FMPEs from the NHANES 2013 to 2014 study population (Fig. 1). The estimated mean number of periodontal sites with CAL3+ based on the analytic NHANES 2013 to 2014 FMPE population is 19.20 sites (95% confidence interval, 18.35–20.05). The relative biases of the Monte Carlo estimates from the FMPE evaluations were less than 1%, as expected (Table 2). Meanwhile, the imputation-based estimators overestimated the FMPE gold standard mean estimates for all RSSMs with percent relative biases increasing as *m*, the number of sampled sites, decreased. Relative bias ranged from 9.5% for RSSM 84 to 16.2% for RSSM 28, which is less than the 25% to 38% bias reported for the population ranking method of selecting PRPs with 14 teeth (i.e., 84 sites; Appendix). Next, the MI-boot variance estimator for the mean number of sites with CAL3+ underestimated the gold standard Monte Carlo variance estimator for RSSMs by 12% to 25% (Fig. 2). Finally, the percent relative efficiency of the MI-boot procedures decreased (with greater information loss) as *m* decreased (Table 2). Specifically, the information loss of RSSM 84 is 12% relative to the FMPE estimator, whereas the loss of RSSM 28 is 55%.

**Table 2.** Evaluation Results for Multiple Imputation–Based Estimators of the Mean Number of Sites with CAL3+ using 500 Simulations of Random Partial-Mouth Periodontal Examinations from NHANES 2013–2014 (*N* = 3,621 Participants).

Site Selection Protocol	Average Estimate^a^	Percent Relative Bias^b^	Average Variance Estimate^a^	Monte Carlo Variance	Percent Relative Efficiency of Mean Extent Estimator^b,c^	Percent Relative Bias of Variance Estimator^d^
FMPE	19.20	−0.02	0.190	0.204	100.4	−6.96
Random 84	21.01	9.45	0.217	0.256	88.1	−15.31
Random 42	21.74	13.23	0.288	0.387	66.3	−25.71
Random 36	21.99	14.55	0.347	0.394	54.9	−11.74
Random 28	22.31	16.20	0.420	0.536	45.5	−21.65

CAL, clinical attachment loss; FMPE, full-mouth periodontal examination.

a The average of the estimates over 500 evaluations.

b The gold standard mean based on the National Health and Nutrition Examination Survey 2013 to 2014 FMPE estimate is 19.201 sites (variance = 0.1909).

c The ratio of the full-mouth variance estimate to the average variance estimate.

d Bias of the average variance estimate relative to the Monte Carlo variance of the estimates of the mean number of sites with CAL3+ (i.e., extent).

### Bias Results for Estimating Prevalence with PRPs

Prevalence estimates per 100 persons from the NHANES 2013 to 2014 FMPE were 90.19 and 83.22 for case definitions of 1 or more and 2 or more sites with CAL3+, respectively. The relative biases of the estimates from the FMPE evaluations were less than 0.1% for both prevalence estimators, confirming good performance of the evaluation methods. All imputation-based RSSM prevalence estimates overestimated the FMPE estimate (Table 3). In particular, relative bias was about 5% for 1 or more sites affected, which compares favorably to the literature (Appendix), and 10% for 2 or more sites.

**Table 3.** Percent Relative Bias of Multiple Imputation–Based Prevalence Estimators Using 500 Simulations of Random Partial-Mouth Periodontal Examinations from NHANES 2013–2014 (*N* = 3,621 Participants).

Site Selection Protocol	1 or More Sites with CAL3+	2 or More Sites with CAL3+
FMPE	0.1	0.1
Random 84	5.3	7.6
Random 42	5.8	9.6
Random 36	5.8	9.7
Random 28	5.8	9.8

The gold standard prevalence based on the National Health and Nutrition Examination Survey (NHANES) 2013 to 2014 FMPE is 90.2% and 83.2% of participants with 1 and 2 or more sites with CAL3+, respectively.

CAL, clinical attachment loss; FMPE, full-mouth periodontal examination.

## Discussion

This study suggests that MI may produce valid estimates for population periodontitis measures from epidemiological surveys using PRPs. Because MI produces complete data sets of full dentitions, MI can surmount the problem of shifting case definitions and sampling protocols over time by allowing reanalyses of periodontitis surveillance data for selected periodontal metrics and sampling protocols (Rozier et al. 2017). Moreover, MI may potentially reduce bias in the association between epidemiological exposures such socioeconomic status or systemic disease indicators and periodontitis in data generated by PRPs (Akinkugbe et al. 2015; Heaton, Garcia, et al. 2018; Alawaji et al. 2022).

In this article, the MI approach gave less biased estimates of periodontitis extent than the standard partial-mouth classification method with established PRPs. While the MI method resulted in 10% relative bias for mean extent measured by CAL3+ for an RSSM with 84 sites, the bias of population rank-based PRPs based on the same number of sites exceeded 25% (Alshihayb et al. 2022). While the ranking method was used to explore and characterize periodontitis misclassification patterns under PRPs, its practicality for periodontitis surveillance is unclear. First, the selection of PRPs is based on the ranking of tooth measures from FMPEs, which may not be available for the population of interest when a PRP is employed in practice. Second, the application of rank-based PRPs to the same data set used to select them may give overly optimistic results in terms of bias and sensitivity than had the selected PRPs been applied to new or “left-out” data. Finally, these authors note that rank-based PRPs tend to overestimate mean severity because they select the most diseased sites.

The MI approach proposed in this article also has limitations. First, misspecification of the imputation model, which is based on a pair of regression models for distributional features of binary outcomes that are clustered in mouths, may lead to invalid results. Because our evaluation was based on a single data set, the true imputation model is unknown. Hence, the source of underestimation of the MI-boot variances in this study is not clear. Future research will employ simulation studies to better illuminate the sources of bias in both estimates and their variances. Second, this article limited consideration to simple periodontal case definitions. Because MI is a general approach, its downstream application to more complex case definitions of periodontal disease such as the 2012 CDC/AAP is possible; however, to obtain estimates with minimal bias, the imputation model would need to account for both CAL and PD. Third, the particular implementation of MI methods was limited to RSSMs. While RSSMs have been extensively studied, FSSMs are the norm in practice.

The use of MI methods in FSSMs or random half-mouth protocols (RHMs) would require that the PRP selects the particular sites and/or teeth needed to estimate the chosen imputation model. For example, the RHM (Drury et al. 1996) that selects opposing contralateral quadrants (i.e., upper right/lower left or upper left/lower right) would not be able to estimate the correlation in our model for “a pair of sites on teeth that are directly above and below each other with 1 tooth on the maxillary jaw and 1 tooth on the mandibular jaw.” On the other hand, a half-mouth PRP that randomly selects 1 upper jaw and 1 lower jaw quadrant without the restriction that they be contralateral would provide the data needed to perform the imputations.

The closest available method to MI for estimating periodontitis prevalence using PRPs is a formula based on the case definition that 1 or more diseased sites meet a threshold for a cardinal measure (e.g., CAL3+; Preisser et al. 2017). The formula, which circumvents the case classification of study participants, is based on a working model for the intensity and pattern of disease in the mouth that assumes disease risk is the same across all tooth sites, and the within-mouth correlation of disease among any 2 sites is constant. Despite the inaccuracy of these assumptions, the formula gave prevalence estimates with bias from 1% to 23% across a range of RSSMs and CAL/PD thresholds. The SDM underlying the formula could be generalized to account for the symmetry of CAL or PD across sites and quadrants (Alshihayb et al. 2022). On the other hand, developing prevalence formulae for more complex case definitions may be challenging.

Given the well-known underestimation bias of the standard method that classifies study participants based only on their partial-mouth data, a statistical model-based approach to estimating periodontitis extent and prevalence using PRPs is advocated. While the formulaic approach of Preisser et al. (2017) and the proposed MI method both require a SDM for each study participant, MI is particularly promising since it can be applied to any downstream case definition once full-mouth data are imputed. NHANES and other surveys have all but abandoned use of PRPs in the past decade. However, development and refinement of the MI approach could conceivably improve PRP accuracy enough to justify, on grounds of logistical efficiency, a return to the regular use of PRPs in large oral epidemiological studies.

## Author Contributions

J.S. Preisser, B.F. Qaqish, contributed to conception and design, data analysis and interpretation, drafted and critically revised manuscript; T. Shing, contributed to conception and design, data acquisition, analysis, and interpretation, drafted and critically revised manuscript; K. Divaris, contributed to conception and design, data interpretation, drafted and critically revised manuscript; J. Beck, contributed to conception, and data interpretation, drafted and critically revised manuscript. All authors gave final approval and agree to be accountable for all aspects of the work.

## Supplemental Material

sj-docx-1-jct-10.1177_23800844221143683 – Supplemental material for Multiple Imputation for Partial Recording Periodontal Examination Protocols Click here for additional data file. Supplemental material, sj-docx-1-jct-10.1177_23800844221143683 for Multiple Imputation for Partial Recording Periodontal Examination Protocols by J.S. Preisser, T. Shing, B.F. Qaqish, K. Divaris and J. Beck in JDR Clinical & Translational Research

## References

[bibr1-23800844221143683] Akinkugbe R Saraiya V Preisser JS Offenbacher S Beck J . 2015. Bias in estimating the cross-sectional smoking, alcohol, obesity and diabetes associations with moderate-severe periodontitis in the Atherosclerosis Risk in Communities study: comparison of full versus partial mouth estimates. J Clin Periodontol. 42(7):609–621. 26076661 10.1111/jcpe.12425 PMC4509916

[bibr2-23800844221143683] Alawaji YN Mostafa N Carvalho RM Alshammari A Aleksejuniene J . 2022. Accuracy and precision of using partial-mouth recordings to study the prevalence, extent and risk associations of untreated periodontitis. Saudi Dent J. 34(2):142–149. 35241904 10.1016/j.sdentj.2021.12.005 PMC8864468

[bibr3-23800844221143683] Alexander AG . 1970. Partial mouth recording of gingivitis, plaque and calculus in epidemiological surveys. J Periodontal Res. 5(2):141–147. 4254169 10.1111/j.1600-0765.1970.tb00707.x

[bibr4-23800844221143683] Alshihayb TS Sharma P Dietrich T Heaton B . 2022. Exploring periodontitis misclassification mechanisms under partial-mouth protocols. J Clin Periodontol. 49(5):448–457. 35246856 10.1111/jcpe.13611

[bibr5-23800844221143683] Bartlett JW Hughes RA . 2020. Bootstrap inference for multiple imputation under uncongeniality and misspecification. Stat Methods Med Res. 29(12):3533–3546. 32605503 10.1177/0962280220932189 PMC7682506

[bibr6-23800844221143683] Beck J Caplan D Preisser J Moss K . 2006. Reducing the bias of probing depth and attachment level estimates using random partial mouth recording. Community Dent Oral Epidemiol. 34(1):1–10. 16423025 10.1111/j.1600-0528.2006.00252.x

[bibr7-23800844221143683] Botelho J Machado V Proença L Mendes JJ . 2020. The new 2018 classification outperforms the 2012 classification regarding the diagnosis and staging of periodontitis on full-mouth PRPs. Sci Rep. 10(1):7093. 32341429 10.1038/s41598-020-63700-6 PMC7184582

[bibr8-23800844221143683] Brand J Buuren S Cessie S Hout W . 2019. Combining multiple imputation and bootstrap in the analysis of cost-effectiveness trial data. Stat Med. 38(2):210–220. 30207407 10.1002/sim.7956 PMC6585698

[bibr9-23800844221143683] Brown LJ Löe H . 1993. Prevalence, extent, severity and progression of periodontal disease. Periodontology 2000. 2:57–71. 10.1111/j.1600-0757.1993.tb00220.x 9673181

[bibr10-23800844221143683] Drury TF Winn DM Snowden CB Kingman A Kleinman DV Lewis B . 1996. An overview of the oral health component of the 1988–1991 National Health and Nutrition Examination Survey (NHANES III–Phase 1). J Dent Res. 75(Suppl 2):620–630. 8594086 10.1177/002203459607502S02

[bibr11-23800844221143683] Eke PI Page RC Wei L Thornton-Evans G Genco R J . 2012. Update of the case definitions for population-based surveillance of periodontitis. J Periodontol. 83(12):1449–1454. 22420873 10.1902/jop.2012.110664 PMC6005373

[bibr12-23800844221143683] Eke PI Thornton-Evans GO Wei L Borgnakke WS Dye BA . 2010. Accuracy of NHANES periodontal examination protocols. J Dent Res. 89(11):1208–2010. 20858782 10.1177/0022034510377793

[bibr13-23800844221143683] Fleiss JL Park MH Chilton NW Alman JE Feldman RS Chauncey HH . 1987. Representativeness of the “Ramfjord teeth” for epidemiologic studies of gingivitis and periodontitis. Community Dent Oral Epidemiol. 15(4):221–224. 3476248 10.1111/j.1600-0528.1987.tb00525.x

[bibr14-23800844221143683] Heaton B Garcia RI Dietrich T . 2018. Simulation study of misclassification bias in association studies employing partial-mouth protocols.J Clin Periodontol. 45(9):1034–1044. 29971808 10.1111/jcpe.12979

[bibr15-23800844221143683] Heaton B Sharma P Garcia RI Dietrich T . 2018. Evaluating periodontal disease misclassification mechanisms under partial-mouth recording protocols. J Clin Periodontol. 45(4):422–430. 29385644 10.1111/jcpe.12874

[bibr16-23800844221143683] Kingman A Albandar JM . 2002. Methodological aspects of epidemiological studies of periodontal diseases. Periodontology 2000. 29:11–30. 10.1034/j.1600-0757.2002.290102.x 12102701

[bibr17-23800844221143683] Kingman A Susin C Albandar JM . 2008. Effect of partial recordings on severity estimates of periodontal disease. J Clin Periodontol. 35(8):659–667. 18513337 10.1111/j.1600-051X.2008.01243.x

[bibr18-23800844221143683] Mills WH Thompson GW Beagrie GS . 1975. Partial-mount recording of plaque and periodontal pockets. J Periodontal Res. 10(1):36–43. 124333 10.1111/j.1600-0765.1975.tb00005.x

[bibr19-23800844221143683] Preisser JS Marks SJ Sanders AE Akinkugbe AA Beck JD . 2017. A new way to estimate disease prevalence from random partial-mouth samples. J Clin Periodontol. 44(3):283–289. 27883200 10.1111/jcpe.12656 PMC5328941

[bibr20-23800844221143683] Preisser JS Qaqish BF . 2014. A comparison of methods for simulating correlated binary variables with specified marginal means and correlations. J Stat Comput Sim. 84(11):2441–2452.

[bibr21-23800844221143683] Prentice RL . 1988. Correlated binary regression with covariates specific to each binary observation. Biometrics. 44(4):1033–1048. 3233244

[bibr22-23800844221143683] Qaqish BF . 2003. A family of multivariate binary distributions for simulating correlated binary variables with specified marginal means and correlations. Biometrika. 90(2):455–463.

[bibr23-23800844221143683] Ramfjord SP . 1959. Indices for prevalence and incidence of periodontal disease.J Periodontol. 30(1):51–59.

[bibr24-23800844221143683] Rozier RG White A Slade GD . 2017. Trends in oral diseases in the U.S. population. J Dent Educ. 81(8):eS97–eS109. 10.21815/JDE.017.016 28765461

[bibr25-23800844221143683] Rubin DB . 1987. Multiple imputation for nonresponse in surveys. New York: Wiley.

[bibr26-23800844221143683] Schomaker M Heumann C . 2018. Bootstrap inference when using multiple imputation: bootstrap inference when using multiple imputation. Stat Med. 37(14):2252–2266. 29682776 10.1002/sim.7654 PMC5986623

[bibr27-23800844221143683] Shafer JL Graham JW . 2002. Missing data: our view of the state of the art. Psychol Methods. 7(2):147–177. 12090408

[bibr28-23800844221143683] Shing TL Preisser JS Zink RC . 2021. GEECORR: a SAS macro for regression models of correlated binary responses and within-cluster correlation using generalized estimating equations. Comput Methods Programs Biomed. 208:106276. 34325377 10.1016/j.cmpb.2021.106276

[bibr29-23800844221143683] Susin C Kingman A Albandar JM . 2005. Effect of partial recording protocols on estimates of prevalence of periodontal disease.J Periodontol. 76(2):262–267. 15974851 10.1902/jop.2005.76.2.262

[bibr30-23800844221143683] Tonetti MS Greenwell H Kornman KS . 2018. Staging and grading of periodontitis: framework and proposal of a new classification and case definition.J Periodontol. 89(Suppl 1):S159–S172. 10.1002/JPER.18-0006 29926952

[bibr31-23800844221143683] Tran DT Gay I Du XL Fu Y Bebermeyer RD Neumann AS Streckfus C Chan W Walji MF . 2014. Assessment of partial-mouth periodontal examination protocols for periodontitis surveillance. J Clin Periodontol. 41(9):846–852. 25041094 10.1111/jcpe.12285 PMC4318801

