# Supplementary material for: Multiple Imputation for Partial Recording Periodontal Examination Protocols
Source: JDR Clin Trans Res. 2023 Jan 16;9(1):52–60. doi: 10.1177/23800844221143683 (PMC10725098; doi:10.1177/23800844221143683)
Supplement: sj-docx-1-jct-10.1177_23800844221143683 – Supplemental material for Multiple Imputation for Partial Recording Periodontal Examination Protocols [file sj-docx-1-jct-10.1177_23800844221143683.docx]

**Supplemental Appendix**

for

“Multiple Imputation for Partial Recording Periodontal Examination Protocols”

by

J.S. Preisser, T. Shing, B.F. Qaqish, K. Divaris, and J. Beck

**Technical details of Step 2 of MI-Boot estimation: imputation of non-selected tooth sites**

Let *i* index the mouth clusters in the *l*-th MI replicate such that *i = 1,…,K*. Suppose that *m_i_* sites were selected from *n_i_* sites in the mouth and there are *n_i_* - *m_i_* tooth sites not selected. We first order the tooth sites for each participant based on their existing teeth with selected sites $Y_{i}^{o}$ listed first and non-selected sites $Y_{i}^{u}$ listed last. Next, generate $Y_{i}^{u}$ from its conditional distribution given $Y_{i}^{o}$ based on its CLF (Qaqish, 2003). To do this, there is no need to explicitly identify the conditional distribution. Rather, we specify the joint distribution for the complete data $Y_{i}^{c}=(Y_{i}^{o},Y_{i}^{u})$ for the *i*-th subject with *n_i_* tooth sites by specifying the mean vector and correlation matrix $(\mu_{i}^{*},R_{i}^{*})$. We then apply a modified CLF algorithm for generating the correlated binary variates sequentially, initially generating $Y_{i,m_{i+1}}$given $Z_{i,m_{i}}=(y_{i1},\ldots,y_{i,m_{i}})'$. In other words, the procedure does not generate $Y_{i}^{o}$ but only generates the unobserved, non-selected values. In summary, since $Y_{i}^{o}$ consists of *m_i_* observed sites, first generate $Y_{i,m_{i+1}|}{(y}_{i1},\ldots,y_{i,m_{i}}$). Next, generate $Y_{i,m_{i+2}}|{(y}_{i1},\ldots,y_{i,m_{i}}$,$Y_{i,m_{i+1}}$) and so on until $Y_{i,n_{i}}{|(y}_{i1},\ldots,y_{i,m_{i}}$,$Y_{i,m_{i+1}}$,…,$Y_{i,n_{i}-1}$) is generated. The formulae for the Bernoulli probabilities for these conditional distributions (Qaqish 2003; Preisser and Qaqish 2014) have been implemented in a SAS macro for the imputations that are available on github: <https://github.com/tshing17/CLF-Imputation-SAS-Macro>.

**Comparison of MI and standard methods for PRPs for estimating mean extent**

Alshihayb et al. (2022) report estimates for mean extent (i.e., average number of teeth meeting specific CAL/PD thresholds) under full-mouth (FM) samples and PRPs using the standard estimation method, which is to classify individuals based on only partial-mouth data (without imputations). Using NHANES 2009-2014 FM examination data that provided PD and CAL on six sites per tooth, they evaluate several PRPs that examine all sites on selected teeth. Percent relative bias (PRB) is calculated from Table 3 of Alshihayb et al. by PRB = (PRP mean extent – FM mean extent)/(FM mean extent)×100. PRB (Table A.1) differs little across measure (CAL, PD) and thresholds (mm). While CPITN and Ramfjord PRPs result in the greatest bias, established half-mouth protocols give about 50% bias and the population ranking methods between 25% and 38% bias. In the ranking methods, FM data is used to select the rank-based PRP and since the same data is used to select the PRP as to evaluate its performance, these estimates may indicate the least possible bias from a PRP with 14 teeth. In practice, the PRP is first selected using FM data external to the PRP, likely resulting in greater bias than in Table A.1. In contrast, the MI method applied to the RSSM PRPs in Table 2 of the main manuscript resulted in 9% bias when selecting 84 sites (equivalent to 84/6 = 14 teeth). Additionally, whereas the Ramfjord PRP based on 6 teeth resulted in 79% bias for CAL, the MI method resulted in 15% bias for 36 sites (equivalent to 36/6 = 6 teeth).

**Table A.1.** Percent relative bias of standard estimate of the mean number of teeth with one or more sites exceeding the indicated threshold (extent, mm) for a selection of PRPs^1^

|  | **PRPs that select up to 14 teeth (when available)** | | | | | | | **PRPs that select fewer teeth** | |
| --- | --- | --- | --- | --- | --- | --- | --- | --- | --- |
| **measure** | **URLL^2^** | **URLR^3^** | **RHM^4^** | **14-teeth^5^ rank-based 1** | **14-teeth^5^ rank-based 2** | **14-teeth^5^ rank-based 3** | **14-teeth^5^ rank-based 4** | **CPITN^6^ (10)** | **Ramfjord^7^ (6)** |
| CAL3+ | -52 | -48 | -50 | -29 | -30 | -34 | -32 | -59 | -79 |
| CAL4+ | -51 | -48 | -50 | -27 | -29 | -30 | -30 | -59 | -79 |
| CAL5+ | -52 | -48 | -50 | -28 | -29 | -28 | -29 | -59 | -79 |
| CAL6+ | -52 | -48 | -50 | -28 | -29 | -27 | -28 | -60 | -79 |
| CAL7+ | -52 | -48 | -50 | -27 | -27 | -25 | -27 | -60 | -79 |
| PD3+ | -53 | -47 | -50 | -30 | -30 | -38 | -31 | -58 | -79 |
| PD4+ | -53 | -47 | -50 | -28 | -28 | -35 | -29 | -53 | -80 |
| PD5+ | -53 | -48 | -51 | -28 | -28 | -35 | -28 | -52 | -81 |
| PD6+ | -54 | -46 | -49 | -29 | -29 | -34 | -29 | -51 | -83 |
| PD7+ | -55 | -45 | -55 | -27 | -27 | -36 | -27 | -55 | -82 |

^1^Percent relative bias is calculated from PRP and full-mouth estimates reported in Table 3 of Alshihayb et al. (2022).^2^ URLL, upper right and lower left; ^3^URLR, upper right and lower right; ^4^RHM, Random Half-Mouth protocol where participants were randomly assigned to a set of opposing contralateral quadrants. ^5^population ranking PRPs are described in Alshihayb et al., respectively as based on the population mean of maximum inter-proximal CAL, the population mean of maximum inter-proximal PD, the population proportion of maximum inter-proximal CAL ≥ 6mm, the population mean of maximum inter-proximal PD≥5mm. ^6^Community Periodontal Index for Treatment Needs, involving ten teeth: #’s 2, 3, 8, 14, 15, 18, 19, 24, 30, 31. ^7^Ramfjord protocol involving 6 teeth: #’s 3, 9, 12, 19, 25 and 28.

**Comparison of MI and standard methods for PRPs for estimating prevalence**

Three articles (Kingman and Albandar, 2002); Susin, Kingman and Albandar, 2005; Eke et al., 2010) report on the underestimation of prevalence by PRPs using the standard estimation method (with classification of individuals based on PRPs and no imputation) using the full-mouth exam as the gold standard. In these data sources, Susin et al. used periodontal examination data from 1,460 dentate persons in Brazil. Kingman and Albander use data on 266 persons with mean age 21.9 whom they describe as having “a fairly high prevalence of periodontal disease due to the manner in which they were selected.” Eke et. al. analyzed 456 persons with mean age 49.6 from NHANES surveys (Table A.2). All papers defined prevalence as 1 or more sites meeting a threshold for CAL. The MI methods report (positive) percent relative bias (PRB) less than 6% in absolute terms based on CAL3+, which is much better than the PRPs shown in Table A.2 with the exception of the RHM methods reported in Susin et al. (2005) that reports -6% (negative bias).

**Table A.2**. Percent relative bias of the standard estimate of prevalence for case definitions based on one or more sites with CAL meeting the indicated threshold (mm) for a selection of PRPs^1^

| **measure** | **Ramfjord^1^** | **RHM^2^** | **NHANES**  **2001^3^** | **NHANES**  **III^3^** |
| --- | --- | --- | --- | --- |
| CAL3+ | -26 | -6 | -30 | -38 |
| CAL4+ | -32 | -9 | - | - |
| CAL5+ | -15 | -10 | - | - |
| CAL6+ | -20 | -17 | -32 | -40 |
| CAL7+ | -23 | -20 | - | - |

^1^Calculated from Table 2 of Kingman and Albandar (2002); ^2^Calculated from Table 1 of Susin, Kingman

and Abandar (2005), using results on 6 sites; ^3^Extracted and rounded from Table 3 of Eke et al. (2010)

**References**

Alshihayb TS, Sharma P, Dietrich T, Heaton B. 2022. Exploring periodontitis misclassification mechansims under

partial-mouth protocols. Journal of Clinical Periodontology 49:448-457.

Eke PI, Thornton-Evans GO, Wei L, Borgnakke WS, Dye BA. 2010. Accuracy of NHANES Periodontal Examination Protocols. J Dent Res. 89(11):1208-2010.

Kingman A, Albandar JM. 2002. Methodological aspects of epidemiological studies of periodontal diseases. Periodontology 2000, Vol. 29, 11–30.

Preisser JS, Qaqish BF. 2014. A comparison of methods for simulating correlated binary variables with specified marginal means and correlations. J Stat Comput Sim. 84(11):2441-2452.

Qaqish BF. 2003. A family of multivariate binary distributions for simulating correlated binary variables with specified marginal means and correlations. Biometrika. 90(2):455-463.

Susin C, Kingman A, Albandar JM. 2005. Effect of Partial Recording Protocols on Estimates of Prevalence of Periodontal Disease. J Periodontol. 76(2):262–267.
